# Novel hormonal therapies in the management of advanced prostate cancer: extrapolating Asian findings to Southeast Asia

**DOI:** 10.1186/s12894-022-01156-8

**Published:** 2023-01-06

**Authors:** Teng Aik Ong, Marniza Saad, Jasmine Lim, Hsien Hooi Lee

**Affiliations:** 1 Department of Surgery, Faculty of Medicine, University of Malaya, Kuala Lumpur, Malaysia; 2 Department of Clinical Oncology, Faculty of Medicine, University of Malaya, Kuala Lumpur, Malaysia; 3 Johnson & Johnson Pte. Ltd., Petaling Jaya, Selangor Malaysia

**Keywords:** Advanced prostate cancer, Southeast Asia, New generation androgen receptor-axis targeted therapies, Abiraterone acetate, Enzalutamide, Apalutamide, Darolutamide

## Abstract

There is a paucity of information on the use of novel hormonal agents in Southeast Asian patients. We reviewed the clinical roles of novel hormonal therapy (NHT), namely abiraterone acetate (AA), enzalutamide, apalutamide and darolutamide, in the management of advanced prostate cancer, and data on its use in Asian patients, in order to extrapolate these findings to the Southeast Asian patient population. There are some differences in the molecular features between the NHTs, which influenced their respective permeabilities through the blood–brain barrier. The Asian sub-analyses of the landmark studies of each NHT were limited. The primary endpoints of the Asian sub-analyses generally reflect the efficacy outcomes of the respective landmark study. Hypertension, fatigue, musculoskeletal disorders, rash, and hot flushes were among the common toxicities observed in Asian patients. Real-world data on AA in the Asian setting is favourable, but data is limited for enzalutamide, apalutamide and darolutamide. Based on the sub-analyses and real-world data, the efficacy and safety of NHTs in the Asian patients showed a similar trend to the respective landmark studies. The lack of clinical trials in the Southeast Asian region hampers the ability to make a robust conclusion on any specific efficacy or safety differences that may be present; clinicians must assume that the broader Asian sub-analyses and real-world data reflects Southeast Asian patients' outcomes.

## Background

Prostate cancer (PCa) is the eighth most common cancer and the eighth most frequent cause of cancer death among men in the South-East Asian region attributing to the aging population [1, 2]. In addition, the mortality-to-incidence ratio of PCa was approximately two times or higher in middle-income Southeast Asian countries compared to high-income Asian countries such as Japan and South Korea [1]. Therefore, understanding of the disease pathophysiology, drug development and accessibility has become increasingly important.

The androgen receptor (AR) axis is an important therapeutic target in the treatment of advanced PCa, through the use of androgen deprivation therapy (ADT) and AR-axis targeted therapies (ARATs) [3]. Abiraterone acetate (AA), enzalutamide, apalutamide or darolutamide are the key novel hormonal therapies (NHTs) which target the AR-axis. They have shown positive results in several clinical trials for the treatment of PCa in different stages (i.e. metastatic castration-naive PCa [mCNPC], nonmetastatic castration-resistant PCa [nmCRPC], and metastatic castration-resistant PCa [mCRPC]) [4–13].

With the increase of PCa burden in Southeast Asia, it is of interest to review the roles of NHT in the management of advanced PCa, NHT availability in Southeast Asia, and data on NHT use in the Asian patients in key clinical trials, especially focusing on the efficacy and adverse events (AEs) in this patient population. Furthermore, we aim to discuss the mechanism of NHT action, as well as to identify the gaps in knowledge on the use of these agents in Southeast Asian patients.

## Methodology

This review was constructed based on key randomised controlled trials (RCTs) and studies covering mCNPC, mCRPC and nmCRPC and treatment with NHT, namely abiraterone acetate (AA), enzalutamide, apalutamide and darolutamide. In September 2021, we conducted a search in the PubMed database to obtain relevant studies published in the English language from 1996 to 2021. Keywords used in the search included the titles of the key trials ("ARAMIS", "ARCHES", "COU-AA-302", "LATITUDE", "PREVAIL", "PROSPER", "SPARTAN", "TITAN"), disease phases ("mCNPC", "mHSPC", "mHNPC", "mCSPC", "mCRPC" and "nmCRPC") and drug names ("abiraterone acetate", "enzalutamide", "apalutamide" and "darolutamide"). The Asian sub-analyses of the specific RCTs were obtained via the keywords "Asian", "Asia", and "sub-analysis". Our search yielded 167,038 hits based on PCa alone, 13,942 hits after refining with specific key words, and 1308 hits after narrowing down articles to Clinical Trial or Randomised Controlled Trial. These articles were then selected and reviewed.

## Review outcomes

### Treatment targets and molecular development of NHTs

The treatment of PCa includes the suppression of androgen synthesis and AR signalling pathway. ADT is the backbone of PCa treatment. However, most patients eventually develop resistance against ADT and progress to castration-resistant PCa (CRPC) [14].

Initially, ligand-based drug design for PCa was based on the structure of testosterone, which is the most prevalent androgen in the body. Since then, many steroidal agonists such as methyltestosterone and oxandrolone and steroidal antagonists such as cyproterone acetate had been approved. Unfortunately, multiple side effects have limited the use of these steroidal agents [15]. Non-steroidal agents such as flutamide, nilutamide and bicalutamide followed, each agent an improvement from the previous agent, although not without their own side effects [16]. Since then, more agents have been developed in order to overcome castration resistance. Several new-generation ARATs have since been developed, which have demonstrated benefit in both metastatic and nonmetastatic disease.

### New-generation AR-axis targeted therapies (ARATs)

Different steps along the AR signalling pathway may be exploited for the treatment of PCa [17]. AA, for one, exerts its effect by inhibiting androgen biosynthesis, while enzalutamide, apalutamide and darolutamide inhibit the AR by blocking its dimerisation, translocation into the nucleus and binding of AR-dimers to the androgen-response elements [17]. Some differences in their mechanism of action, efficacy and adverse effects may be attributed to the chemical structures and molecular differences between these drugs. The molecular differences between the new-generation ARATs are shown in Table 1.

**Table 1:** Molecular differences between the new-generation ARATs

	Abiraterone acetate	Enzalutamide	Apalutamide	Darolutamide
Molecular structure [54–57]				
Ability to cross the blood–brain barrier [19, 34]	Yes			
AR inhibition (AR-HEK293 cells) [34]	n.a.	Full antagonist (IC_50_ = 219 nM)	Full antagonist (IC_50_ = 200 nM)	Full antagonist (IC_50_ = 26 nM)
Molecular weight [54–57]	391.5	464.44	477.4	398.8
pK_a_ of molecule [57–60]	5.19	No pK_a_ between pH 3–11	9.7	11.75
Computed LogP [54–57]	5.2	3.6	3	1.8
Activation of mutant AR [34]	n.a.	Yes (7877L)	Yes (7877L)	None described
Packing size [60–63]	250 mg and 500 mg tablets	40 mg capsules	60 mg tablets	300 mg tablets
Recommended dosage [60–63]	1000 mg/day	160 mg/day	240 mg/day	1200 mg/day
Availability in SEA countries (as of January 2022)	Malaysia, Singapore, Indonesia, Thailand, Philippines, Vietnam	Malaysia, Singapore, Indonesia, Thailand, Philippines	Malaysia, Singapore, Indonesia, Thailand	Singapore

*AR* androgen receptor, *BD* twice daily, *IC*_*50*_ half maximal inhibitory concentration, *n.a.* not applicable, *OD* once daily, *SEA* Southeast Asia

#### Abiraterone acetate (AA)

AA is a potent inhibitor of CYP17A1, which is the key enzyme involved in the synthesis of androgen including testosterone [17, 18]. The inhibition of testosterone synthesis not only occurs in the testes, but also in the adrenal glands and PCa cells [17]. AA is associated with side effects related to mineralocorticoids excess leading to hypokalaemia, fluid retention, or hypertension, which necessitates its combination with corticosteroids to reduce these side effects [19].

Several phase 3 trials have been performed with AA in the treatment of different phases of advanced PCa, namely mCNPC and mCRPC, with positive outcomes. The Asian sub-analysis on AA efficacy is only available from the mCNPC trial (LATITUDE), as summarised in Table 2. No Asian sub-analysis for the COU-AA-301 and COU-AA-302 trials (AA in post-chemotherapy and chemotherapy-naive mCRPC patients, respectively) was available. The safety outcomes of AA in Asian patients are summarised in Table 3.

**Table 2:** Efficacy outcomes of phase 3 trials evaluating NHTs (Asian sub-analyses and the landmark studies)

Agent	Disease phase	Asian study population (n)	Primary endpoint(s)	Outcomes (Asian study)	Outcomes (Landmark study)
Abiraterone acetate	mCNPC	LATITUDE^a^ Japanese subgroup analysis (n = 70) [36, 37]	OS, rPFS	Median follow-up time: 56.6 months (range 2.5–64.2) Median OS NR Overall 5-year survival rate: 69.2% (AAP) versus 53.7% (placebo) Median rPFS: NR (AAP) versus 22 months (placebo) (HR 0.219; 95% CI 0.086–0.560)	Median follow-up time: 51.8 months (interquartile range 47.15–57.03) Median OS: 53.3 months (95% CI 48.2–NR) (AAP) versus 36.5 (95% CI 33.5–40.0) months (placebo) (HR 0.66; 95% CI 0.56–0.78; *p* < 0.0001) [5] Median rPFS was 33 months (AAP) versus 14.8 months (placebo); (HR 0.47; 95% CI 0.39–0.55; *p* < 0.001) [5]
Enzalutamide	mCNPC	ARCHES^b^ Japanese subgroup analysis (n = 92) [38]	rPFS	Median follow-up time: 15.7 months (enzalutamide) versus 15.5 months (placebo) Risk of radiographic progression or death reduced by 61% versus placebo (HR 0.39; 95% CI 0.13–1.18)	Median follow-up time: 44.6 months Survival extended versus placebo (HR 0.66; 95% CI 0.53–0.81; *p* < 0.0001) [8]
	nmCRPC	PROSPER^c^ subgroup analysis by region (Asia vs. North America) [39]	OS	Median follow-up time: Not reported OS benefit similar across Asia and North America (HR 1.164 [95% CI 0.824, 1.644; *p* = 0.3883])	Median follow-up time: ~ 48 months Median OS: 67.0 months (95% CI 64.0-NR) (enzalutamide) versus 56.3 months (95% CI 54.4–63.0) (placebo) [10]
	mCRPC	PREVAIL^d^ Asian subpopulation (Japan, Korea, and Singapore) (n = 148) [40]	rPFS, OS	Median follow-up time: Not reported rPFS HR 0.38 (95% CI 0.10–1.44) OS HR 0.59 (95% CI 0.29–1.23) OS not reached	Median follow-up time: 69 months Median OS: 36 months (95% CI 34–38) (enzalutamide) versus 31 months (95% CI 29–34) (placebo) [11]
Apalutamide	mCNPC	TITAN^e^ Japanese subgroup analysis (n = 51) [42]	OS, rPFS	Median follow-up time for OS: 25.72 months (range 5.8–31.4) OS: HR 0.840 (95% CI 0.210–3.361; *p* = 0.805) Median follow-up time for rPFS: 22.05 months (range 1.1–29.3) rPFS: HR 0.712 (95% CI 0.205–2.466; *p* = 0.59)	Median follow-up time: 44.0 months Median OS NR (apalutamide) versus 52.2 months (placebo) (HR 0.65; 95% CI 0.53–0.79; *p* < 0.0001) [12] rPFS: HR 0.48 (95% CI 0.39–0.60; *p* < 0.001) [12] 24-month rPFS: 68.2% (apalutamide) versus 47.5% (placebo) (HR 0.484; 95% CI 0.391–0.600; *p* < 0.001) [41]
		TITAN^e^ East Asia subgroup analysis (China, Japan and Korea) (n = 62) [41]	rPFS, OS	Median follow-up time: 21.2 months (range 1.0–33.0) (apalutamide) versus 20.3 months (range 4.6–32.1) (placebo) 24-month rPFS: 76.1% (apalutamide) versus 52.3% (placebo) (HR 0.506; 95% CI 0.302–0.849; *p* = 0.009)	
	nmCRPC	SPARTAN^f^ Asian subpopulation (Japan, Taiwan, and South Korea) analysis (n = 126) [44]	MFS, PSA	Median follow-up time: Not reported MFS improvement was similar for Asian patients (HR 0.29; *p* < 0.001) and non-Asian patients (HR 0.28; *p* < 0.0001) PSA response: 82% (Asian patients) versus 91% (non-Asian patients)	Median follow-up time: Not reported Patients with PSA not declined to < 0.2 ng/mL had a 54% risk reduction for MFS (HR 0.46; 95% CI 0.37–0.57; *p* < 0.0001), whereas patients with PSA that declined to < 0.2 ng/mL had an 88% risk reduction for MFS (HR 0.12, *p* < 0.0001) [43] Median follow-up time: 52.0 months PSA progression: HR 0.07 (95% CI 0.06–0.09; 95% CI 0.08–0.17; nominal *p* < 0.0001) [13]
		SPARTAN^f^ Japanese subpopulation analysis (n = 55) [45]	MFS	Median treatment duration: 5.7 months (apalutamide) versus 11.0 months (placebo) Median MFS: NR (95% CI 10.97-NE) (apalutamide) versus 18.23 months (95% CI 11.04–18.50) (placebo)	
Darolutamide	nmCRPC	ARAMIS^g^ Japanese subpopulation analysis (n = 95) [47]	MFS	Median treatment duration: 14.8 months (darolutamide) versus 10.9 months (placebo) Median MFS: NR (darolutamide) versus 18.2 months (placebo) (HR 0.28; 95% CI 0.11–0.70)	Median follow-up time: 29.0 months [6] Median MFS: 40.4 months (darolutamide) versus 18.4 months (placebo) (HR 0.41; 95% CI 0.34–0.50; 2-sided *p* < 0.0001) [46]

*AAP* abiraterone acetate plus prednisolone, *CI* confidence interval, *HR* hazard ratio, *mCNPC* metastatic castration-naive PCa, *mCRPC* metastatic castration-resistant PCa, *MFS* metastasis-free survival, *NHT* novel hormonal therapy, *nmCRPC* non-metastatic castration-resistant PCa, *NR* not reached, *OS* overall survival, *PSA* prostate-specific antigen, *rPFS* radiographic progression-free survival

^a^LATITUDE is the study of AA plus low-dose prednisolone plus ADT versus ADT alone in newly diagnosed participants with high-risk mCNPC. ^b^ARCHES is the study of ADT with enzalutamide or placebo in men with mCNPC. ^c^PROSPER is the study of enzalutamide in men with nmCRPC. ^d^PREVAIL is the study of enzalutamide in men with mCRPC. ^e^TITAN is the study of apalutamide in men with mCNPC. ^f^SPARTAN is the study of apalutamide in men with nmCRPC. ^g^ARAMIS is the study of darolutamide in men with nmCRPC

**Table 3:** Safety outcomes of phase 3 clinical trials evaluating NHTs (Asian sub-analyses and the landmark studies)

Agent	Disease phase	Asian study population (n)	AEs > 10% (Asian study)	AEs grade 3–5 (Asian study)	Total AEs grade 3–5 (Asian study)	Total AEs grade 3–5 (Landmark study)	Discontinuation due to AEs (Asian study)	Deaths due to AEs (Asian study)
Abiraterone acetate	mCNPC	LATITUDE^a^ Japanese subgroup analysis (n = 70) [36, 37]	Hypertension (51.4%) Hypokalaemia (42.9%) Nasopharyngitis (40.0%) Weight increased (34.3%) Hot flush (31.4%) Back pain (28.6%) ALT increased, AST increased (25.7% each) Hyperglycaemia (22.9%) Rib fracture, insomnia, influenza (14.3% each) Constipation, dental caries, diarrhoea, vomiting, haematuria, hyperbilirubinaemia (11.4% each)	Hypertension (34.3%) Hypokalaemia (14.3%) Hyperglycaemia (11.4%) ALT increased, AST increased Dental caries, Diarrhoea, Bone pain (2.9% each)	68.6% (AAP) versus 25.7% (placebo)	68% (AAP) versus 50% (placebo) [5]	AAP group 8.6%; placebo-AAP crossover group 33.3%; placebo 11.4%	AAP 5.7%; placebo-AAP crossover none; placebo none
Enzalutamide	mCNPC	ARCHES^b^ Japanese subgroup analysis (n = 92) [38]	Hot flushes (27.8%) Nasopharyngitis (25.0%) Hypertension (19.4%) Abnormal hepatic function, fractures, musculoskeletal events (13.9% each) Rash (11.1%)	Hypertension (13.9%) Abnormal hepatic function, fractures (5.6% each) Increased weight, convulsion, decreased neutrophil count, ischaemic heart disease, loss of consciousness (2.8% each)	47% (enzalutamide) versus 25% (placebo)	Not reported	Enzalutamide 11.1%; placebo 1.8%	None
	nmCRPC	PROSPER^c^ subgroup analysis by region (Asia vs. North America) [39]	Not reported	Not reported	Not reported	48% (enzalutamide) versus 27% (placebo) [10]	Not reported	Not reported
	mCRPC	PREVAIL^d^ Asian subpopulation (Japan, Korea, and Singapore) (n = 148) [40]	Fatigue, decreased appetite (20.5% each) Constipation (17.8%) URTI, falls (15.1% each) Back pain (13.7%) Pollakiuria (12.3%) Nausea (11.0%)	Back pain (2.7%) Fatigue (1.4%)	31.5% (enzalutamide) versus 22.7% (placebo)	53% (enzalutamide) versus 38% (placebo) [11]	Enzalutamide 4.1%; placebo 5.3%	Not reported
Apalutamide	mCNPC	TITAN^e^ Japanese subgroup analysis (n = 51) [42]	Skin rash (50.0%) Viral upper respiratory tract infection (39.3%) Hot flush (32.1%) Pruritus, weight increased (25.0% each) Hypertension (17.9%) Fracture, pyrexia, constipation (14.3% each) Fall, upper respiratory tract infection, fatigue, nausea, stomatitis, leukopenia, arthralgia, injection site induration (10.7% each) Hypothyroidism (7.1%)	Fracture, fall (13.0% each) Hypertension (10.7%) Skin rash (8.7%) Weight increased (3.6%)	42.9% (apalutamide) versus 39.1% (placebo)	7.6% (apalutamide) versus 2.7% (placebo) [12]	Apalutamide 7.1%; placebo 4.3%	Apalutamide none; placebo none
		TITAN^e^ East Asia subgroup analysis (China, Japan and Korea) (n = 62) [41]	Rash (37.3%) Hypertension (22.7%) Weight increased (21.8%) Weight decreased (18.2%) Pruritus (17.3%) Hot flush (16.4%) Upper respiratory tract infection (14.5%) Viral upper respiratory tract infection (12.7%) Arthralgia (12.7%) Constipation (11.8%) Pain in arm or leg (10.9%) Rash, generalised (10.0%)	Hypertension, rash (10.9% each) Weight increased (3.6%) Weight decreased, upper respiratory tract infection, fracture (0.9% each)	40.9% (apalutamide) versus 38.2% (placebo)		Apalutamide 7.3%; placebo 4.5%	Apalutamide none; placebo 2.7%
	nmCRPC	SPARTAN^f^ Asian subpopulation (Japan, Taiwan, and South Korea) analysis (n = 126) [44]	No meaningful differences between Asian versus non-Asian patients, except for rash (38% vs. 22%)	Not reported	39% (apalutamide) versus 46% (placebo)	56% (apalutamide) versus 36% (placebo) [13]	Asian patients 15%; non-Asian patients 10%	Not reported
		SPARTAN^f^ Japanese subpopulation analysis (n = 55) [45]	Skin rash (56%)	Rash maculopapular (5.9%) Hydronephrosis, rash macular, rash generalised, drug eruption, miliaria, presyncope, spinal cord compression, thrombotic cerebral infarction, decreased appetite, hyperkalaemia, lumbar spinal stenosis, lumbar vertebral fracture, hypertension, pleural effusion, pneumonia aspiration, weight decreased, amylase increased, anaemia, malignant neoplasm of renal pelvis, renal impairment, cystitis haemorrhagic, cardiac failure, cataract, prostatitis (2.9% each)	44.1% (apalutamide) versus 23.8% (placebo)		Apalutamide 20.6%; placebo 9.5%	Apalutamide none; placebo 4.8%
Darolutamide	nmCRPC	ARAMIS^g^ Japanese subpopulation analysis (n = 95) [47]	Constipation, falls (12.9% each) Fracture (11.3%)	Bladder neoplasm, hydronephrosis (3.2% each) Abscess, ALT increased, anaemia, angina pectoris, arrhythmia, AST increased, asthma, bronchitis, cataract, colon cancer, decreased appetite, fall, fracture, gingivitis, haematuria, hepatic function abnormality, influenza, iron deficiency anaemia, neutropenia, neutrophil count decreased, pancreatic carcinoma, pneumonia, postoperative ileus, pulmonary mass, rectal cancer, urinary retention (1.6% each)	43.5% (darolutamide) versus 48.5% (placebo)	Not reported	Darolutamide 8.1%; placebo 6.1%	Not reported

*AAP* abiraterone acetate plus prednisolone, *AE* adverse event, *ALT* alanine aminotransferase, *AST* aspartate aminotransferase, *mCNPC* metastatic castration-naive PCa, *mCRPC* metastatic castration-resistant PCa, *NHT* novel hormonal therapy, *nmCRPC* non-metastatic castration-resistant PCa

^a^LATITUDE is the study of AA plus low-dose prednisolone plus ADT versus ADT alone in newly diagnosed participants with high-risk mCNPC. ^b^ARCHES is the study of ADT with enzalutamide or placebo in men with mCNPC. ^c^PROSPER is the study of enzalutamide in men with nmCRPC. ^d^PREVAIL is the study of enzalutamide in men with mCRPC. ^e^TITAN is the study of apalutamide in men with mCNPC. ^f^SPARTAN is the study of apalutamide in men with nmCRPC. ^g^ARAMIS is the study of darolutamide in men with nmCRPC

In a real-world data (RWD) study performed for mCRPC in the Southeast Asian cohort, 93 patients were treated with AA plus prednisolone (AAP) in Thailand and Malaysia (58.1% and 41.9% of patients, respectively). Patients were treated for a median duration of 10 months (IQR 5.6–17.1). Malaysian patients had numerically shorter median overall survival (OS) and biochemical progression-free survival (bPFS) (OS 17.8 months, 95% CI 6.4–29.1; bPFS 10.4 months, 95% CI 8.8–12.0) versus Thai patients (OS 27.0 months, 95% CI 11.3–42.7; bPFS 14.0 months; 95% CI 5.8–22.2), although the differences were non-significant (both *p* > 0.05). Patients who received > 10 months of AAP treatment had lower risk of death and longer bPFS, compared to patients who received ≤ 10 months treatment duration (HR 0.10, 95% CI 0.05–0.22 and HR 0.13, 95% CI 0.06–0.25, respectively). AAP was well-tolerated in the Southeast Asian cohort with comparable survival benefits to other Asian populations. Lower PSA levels at AAP initiation, positive prostate-specific antigen (PSA) response, and chemotherapy-naive were significant factors in deciding the duration of AAP treatment [20].

Another retrospective study was performed in a single Chinese centre involving 60 chemotherapy-naive mCRPC patients treated with AAP (n = 43) or prednisolone alone (n = 17). The median follow-up time was 14.0 months (range 7.0–18.5 months). The AAP group had significantly longer median PSA progression-free survival (PFS) (10.3 vs. 3.0 months, *p* < 0.001), rPFS (13.9 vs. 3.9 months, *p* < 0.001), and OS (23.3 vs. 17.5 months, *p* = 0.016) compared to the prednisolone group. The AAP and prednisolone groups had elevated alanine aminotransferase as the most frequently reported grade 3/4 adverse event, at 11.6% and 11.8% of patients, respectively. The AEs had not caused treatment cessation in patients. A short duration (≤ 18 months) from ADT to castration resistance was a significant factor associated with shorter OS (*p* = 0.007). AA was reported to have a favourable safety and efficacy profile for the treatment of chemotherapy-naive mCRPC patients of Asian descent [21].

Similarly, in a retrospective analysis performed on 200 mCRPC patients treated with AAP at four tertiary hospitals in Singapore (81.5% of the patients were chemotherapy-naïve and 18.5% were post-chemotherapy), median OS was 20.0 (95% CI 18.3–22.9) and 10.5 (95% CI 1.1–40.5) months for chemotherapy-naïve and post-chemotherapy cohorts, respectively. A subset analysis (n = 108) showed that chemotherapy-naïve patients who had prior ADT for > 12 months had significantly longer OS (*p* < 0.001). Incidence of grade 3/4 events were around 6.6%, with the most common side effect being hypertension (2.4%) [22].

In a real-world clinical experience with AA in mCRPC treatment in Hong Kong, 110 mCRPC patients were treated with AAP (58 chemotherapy-naïve and 52 post-chemotherapy patients). The median follow-up time was 7.5 versus 11.4 months for chemotherapy-naïve versus post-chemotherapy patients, respectively. The median OS and PFS were 18.1 versus 15.5 months and 6.7 versus 6.4 months for chemotherapy-naïve versus post-chemotherapy patients, respectively. Chemotherapy-naïve patients who had visceral diseases had significantly inferior OS (2.8 vs. 18.0 months, *p* = 0.0007) and PFS (2.8 vs. 6.8 months, *p* = 0.0088) versus those without. Pain control was comparable in both chemotherapy-naïve and post-chemotherapy groups. The most common toxicities grade ≥ 3 were hypertension (6.9% vs. 5.8%) and hypokalaemia (3.4% vs. 3.8%) in chemotherapy-naïve versus post-chemotherapy patients. Positive PSA response (≥ 50% drop of PSA from baseline) within the first 3 months of therapy was associated with favourable OS and PFS in both chemotherapy-naïve and post-chemotherapy groups [23].

In a retrospective RWD analysis to evaluate the efficacy of AA in 51 Turkish mCRPC patients, 64.7% received AA post-chemotherapy and 35.3% were chemotherapy-naïve. The median OS was 17.3 months (range 9.3–33.1). Median OS was 12.7 months (range 9.4–18.3) and 29.4 months (range 9.3–33.0) in the chemotherapy-naïve and post-chemotherapy groups, respectively (*p* = 0.236). In addition, median rPFS was 10.1 months (range 4.5–18.4). rPFS was 10.1 months (IQR 6.0–14.7) in the chemotherapy-naïve group and 9.7 months (range 4.0–18.4) in the post-chemotherapy group (*p* = 0.808). PSA-PFS was 7.4 months (range 4.6–13.4) in the chemotherapy-naïve group and 9.1 months (range 4.8–13.1) in the post-chemotherapy group (*p* = 0.843). AA was considered as an effective and reliable agent in this study [24].

In a study on low dose abiraterone taken with food, 36 patients with progressive CRPC from seven centres in the United States and Singapore were randomly assigned to receive either standard dose AA (STD; 1000 mg fasting) or low-dose AA (LOW; 250 mg with a low-fat meal). Both arms also received prednisolone 5 mg twice daily. After 12 weeks, a greater effect was observed on PSA in the LOW group (mean log change, 21.59) versus STD group (21.19). The PSA response rate was 58% and 50% in LOW and STD, respectively. The median PFS was ~ 9 months in both groups, and androgen levels showed similar reduction in both arms. AA concentrations was higher in STD, but there was no difference in PSA response or PFS between the two groups. Low-dose AA with low-fat meal was found to be noninferior to standard dosing with respect to PSA metrics [25].

#### Enzalutamide

Enzalutamide competitively binds to the AR ligand-binding domain and subsequently inhibits its translocation to the nucleus and binding to DNA [26]. In addition, enzalutamide has also been shown to decrease proliferation and increase PCa cell death. It results in tumour regression in castration-resistant xenograft models, and was reported to show persistent in vitro activity in the setting of AR splice variants such as AR-V7 [27].

Positive outcomes of enzalutamide were observed in several phase 3 trials for different PCa phases (mCNPC, nmCRPC and mCRPC). The Asian sub-analyses of these clinical trials in regards to efficacy and safety are listed in Tables 2 and 3, respectively. The AFFIRM study (enzalutamide in mCRPC patients post-docetaxel) is not included, as no Asian sub-analysis have been made available.

A retrospective real-world study evaluated the medical records of 199 Korean chemotherapy-naive mCRPC patients who received enzalutamide daily. Out of this, 89 patients also received concurrent ADT. Over 80% of the patients had a Gleason score of ≥ 8, and one-third of patients had European Cooperative Oncology Group Performance Status (ECOG PS) 0, with an overall mortality rate of 12%. Median OS was not achieved, and 76.7% of patients were alive at 30 months. Median time to PSA progression was 6 months. Patients receiving concurrent ADT achieved significantly higher OS rate at 2 years and significantly longer duration of PSA PFS versus those receiving enzalutamide alone (84.6% vs. 71.7% [*p* = 0.015] and 8.0 months vs. 4.6 months [*p* = 0.008], respectively). The incidence of adverse events of grade ≥ 3 was 1.7%. ADT administered concurrently with enzalutamide was shown to improve OS significantly (HR 0.346, 95% CI 0.125–0.958) [28].

The efficacy, safety, and pharmacokinetics of enzalutamide was evaluated in Japanese CRPC patients; 14 patients received enzalutamide at a standard dose (160 mg/day) or reduced doses (80 or 120 mg/day). The PSA, AEs, and steady-state trough plasma concentrations of enzalutamide (C_SS, ENZ_) and its active metabolite (N-desmethyl enzalutamide [NDE])(C_SS, NDE_) were then analysed. Ninety-two percent of patients achieved a PSA drop of ≥ 50% from baseline. AEs were few and mild. The study reported no differences in C_SS, ENZ_ and C_SS, NDE_ between the study subjects and other ethnic groups from previous studies. Enzalutamide was deemed adequately effective and safe in Japanese CRPC patients, even at reduced doses [29].

A retrospective real-world study of first and later lines of enzalutamide in mCRPC patients in Hong Kong revealed an overall PSA response rate of 43.6%; PSA response rates were 73.5%, 35.1%, and 19.2% for first-line (1L), second-line (2L), and third-/fourth-line (3L and 4L) treatment, respectively. PFS and OS were significantly associated with the line of treatment in the univariate survival analysis (1L/2L/3L and 4L; PFS, 7.1/3.9/2.2 months; OS, not reached/15.8/7.4 months; both *p* = 0.0002), but not in the multivariate analysis. Fatigue (grade 1/2, 54.7%; grade 3/4, 9.4%) was elevated compared to AFFIRM (any grade, 34%) and PREVAIL (any grade, 36%) trials. 3L or 4L treatment was significantly associated with grade 2 fatigue. This study concluded that earlier lines of enzalutamide treatment were associated with longer PFS and OS, more frequent PSA response, and less fatigue [30].

No RWD study was found on the use of enzalutamide in advanced PCa patients in Southeast Asia.

#### Apalutamide

Apalutamide has a 7–10-fold higher affinity to the AR ligand-binding domain versus bicalutamide, which impedes AR dimer translocation into the nucleus [14]. The structural difference between apalutamide and enzalutamide lies on the cyclisation of the dimethyl moiety in enzalutamide into a cyclobutyl moiety in apalutamide, as well as the substitution of carbon in enzalutamide's 2-cyanophenyl moiety to nitrogen in apalutamide's 2-cyanopyridine moiety, which increases the electron density and introduces heterocylisation of the ring [31]. These structural changes contributed to the lower LogP value of apalutamide compared to enzalutamide, indicating increased hydrophilicity which affects its permeability through the blood–brain barrier [32]. In LNCaP xenograft mice, the brain-plasma concentration ratio of apalutamide was shown to be fourfold lower than enzalutamide after 28 days of treatment, suggesting apalutamide's lower threshold for clinical seizures and central nervous system (CNS) toxicities [33].

Phase 3 trials have been reported with apalutamide in mCNPC and nmCRPC patients. The Asian sub-analyses of apalutamide clinical trials are shown in Table 2 (efficacy outcomes) and Table 3 (safety outcomes).

No RWD study was found on the use of apalutamide in advanced PCa patients in Asia.

#### Darolutamide

Similar to enzalutamide and apalutamide, darolutamide blocks the AR dimer translocation into the cell nucleus. However, darolutamide has a distinct molecular structure compared to enzalutamide and apalutamide [34]. The main differentiating feature of darolutamide versus enzalutamide and apalutamide is the opening of the ring into a long hydrocarbon chain, making it the most hydrophilic of the three molecules [35]. Comparison of darolutamide versus enzalutamide in blood–brain barrier (BBB) penetration by using ^14^C-labeled whole-body autoradiography in an animal model showed that darolutamide has a tenfold lower BBB penetration compared to enzalutamide, which may translate to fewer CNS adverse effects such as falls [34, 35]. Darolutamide also has a higher AR inhibition potency versus enzalutamide and apalutamide, and does not activate AR mutants such as AR(F877L), AR(W742L) and AR(T878A) [34].

A phase 3 trial has been performed with darolutamide for the treatment of nmCRPC patients. The Asian sub-analysis of this clinical trial was compared with the landmark study in Table 2. Safety outcomes are shown in Table 3.

No RWD study was found on the use of darolutamide in advanced PCa patients in Asia.

## Discussion

Overall, the Asian sub-analyses reflect the outcomes of its respective landmark studies. In LATITUDE, Japanese mCNPC patients on AA achieved higher 5-year survival rate compared to patients receiving placebo, reflecting the findings reported in the landmark study, in which median OS was significantly longer with AA versus placebo [5, 36]. In addition, the median radiographic progression-free survival (rPFS) was not reached with AA versus 22 months with placebo; the efficacy outcomes in the Japanese subpopulation were deemed consistent with the landmark study [37] (Table 2). A number of AEs were frequently observed in Japanese patients receiving AA, the most common of which includes hypertension, hypokalaemia and nasopharyngitis [36]. The frequency of grade ≥ 3 AEs was similar between the AA groups in the Japanese sub-analysis (68.6%) and the landmark study (68%), although grade ≥ 3 AEs were twice as frequent in patients receiving placebo in the landmark study (50%) compared to the Japanese sub-analysis (25.7%) [5, 36] (Table 3). RWD studies involving Asian mCRPC patients showed positive treatment outcomes with AA, and AA was well-tolerated [20–25]. The most common toxicities with AA observed in real-world studies include hypertension and hypokalaemia [22, 23]. No real-world study on AA involving Asian patients were found for mCNPC.

In ARCHES, Japanese mCNPC patients on enzalutamide showed lower risk of radiographic progression or death compared to placebo; these outcomes were similar to the landmark study, in which survival was extended with enzalutamide versus placebo [8, 38]. For nmCRPC patients on enzalutamide, the landmark PROSPER study reported longer median OS achieved with enzalutamide compared to placebo; similarly, a PROSPER subgroup analysis by region reported that OS benefit with enzalutamide was similar across Asia and North America [10, 39]. As for mCRPC patients on enzalutamide, the PREVAIL Asian sub-analysis involving patients from Japan, Korea and Singapore reported improved rPFS and OS compared to placebo [40]. Similarly, a longer median OS was achieved for mCRPC patients on enzalutamide versus placebo in the landmark PREVAIL study [11] (Table 2). The most common AEs observed with enzalutamide in the Asian sub-analyses vary across mCNPC or mCRPC patient types (data on the most common AEs among nmCRPC patients were not available); hot flushes, nasopharyngitis and hypertension were some of the common AEs seen in mCNPC patients, whereas fatigue, decreased appetite and constipation were seen in mCRPC patients. Fewer Asian mCRPC patients in the PREVAIL sub-analysis (31.5%) had AEs grade ≥ 3 compared to the landmark study (53%) [11, 40] (Table 3). In real-world studies carried out in Korea and Japan, treatment with enzalutamide yielded beneficial outcomes and were well-tolerated [28, 29].

mCNPC patients receiving apalutamide in the landmark TITAN study reported that the median OS was not reached with apalutamide versus 52.2 months with placebo (*p* < 0.0001) [12]. The hazard ratio for rPFS was 0.48 for apalutamide versus placebo (*p* < 0.001), and the 24-month rPFS was also higher with apalutamide versus placebo (*p* < 0.001) [41]. The TITAN Japanese subgroup analysis also reported improved OS and rPFS with apalutamide (hazard ratios both < 1), similar to the overall population [42]. In addition, the TITAN East Asia subgroup analysis involving patients from China, Japan and Korea also showed improved 24-month rPFS with apalutamide versus placebo (*p* = 0.009) [41]. On the other hand, the landmark SPARTAN study reported that nmCRPC patients receiving apalutamide whose PSA declined to < 0.2 ng/mL showed a larger risk reduction in metastasis-free survival (MFS) than patients whose PSA did not decline to < 0.2 ng/mL (*p* < 0.0001) [43]. In addition, apalutamide also tremendously reduced the risk of PSA progression compared to placebo (*p* < 0.0001) [13]. In the Asian sub-analysis of SPARTAN involving patients from Japan, Taiwan and South Korea, MFS improvement with apalutamide was reported to be similar between Asian patients and non-Asian patients versus placebo (both *p* < 0.0001) [44]. However, PSA response was slightly lower with Asian patients (82%) compared to non-Asian patients (91%) [44]. The Japanese sub-analysis of SPARTAN reported that median MFS was not reached, compared to 18.23 months with placebo [45] (Table 2). Rash was a significant AE reported with apalutamide in the Asian sub-analyses of TITAN and SPARTAN [41, 42, 44, 45]. More patients were reported to have AE grade ≥ 3 in the TITAN Japanese and Asian (China, Japan and Korea) sub-analyses (42.9% and 40.9%, respectively) compared to the landmark TITAN study (7.6%) [12, 41, 42]. The difference in the proportion of patients with AEs grade ≥ 3 was less pronounced in the SPARTAN Japanese and Asian (Japan, Taiwan and South Korea) sub-analyses (44.1% and 39%, respectively) compared to the landmark SPARTAN study (56%) [13, 44, 45] (Table 3). No RWD study was found on the use of apalutamide in advanced PCa patients in the Asian population.

In the landmark ARAMIS study involving nmCRPC patients, median MFS was significantly longer with darolutamide compared to placebo (*p* < 0.0001) [46]. Similar outcomes were observed in the Japanese sub-analysis of ARAMIS, with median MFS not reached with darolutamide versus 18.2 months with placebo [47] (Table 2). Common AEs observed in the Asian sub-analysis were constipation, falls and fractures; fewer grade ≥ 3 AEs were observed with darolutamide (43.5%) compared to placebo (48.5%) [47] (Table 3). Similar to apalutamide, no RWD study was found on the use of darolutamide in advanced PCa patients in the Asian population.

Generally, hypertension, musculoskeletal disorders, rash, nasopharyngitis, hot flushes, falls and fractures are among the common AEs observed with NHT in Asian patients (all grades). The most common Grade ≥ 3 AEs include hypertension and fractures, among others (Table 3). However, primary outcomes in several Asian sub-analyses were not reached, and certain safety outcomes were not reported.

A lack of clinical trial analysis performed in the Southeast Asian region leads to uncertainty in understanding how the hormonal therapies impact the Southeast Asian PCa population. This raises a question: how does the Southeast Asian data compare with the rest of the world? The Asia Pacific Advanced Prostate Cancer Consensus Conference (APAC APCCC) 2018, for example, had discussed the increased toxicity of docetaxel in Asian patients compared to Caucasian patients [48].

Currently, clinicians can only extrapolate findings from clinical trial sub-analyses and real-world evidences from the broader Asian region to Southeast Asia. However, many Asian sub-analyses of major NHT studies were focused on East Asian ethnicities, particularly the Japanese population. The relevance of these Asian sub-analyses to specifically Southeast Asian patients may be limited, owing to the homogeneity of the Japanese patients. Southeast Asian populations are heterogeneous, consisting of multiple ethnicities and countries. Therefore, development of Southeast Asian sub-analyses of major NHT trials would enable clinicians from this region to understand the expected outcomes when treating patients and make informed choices. We also propose future clinical trials to be more inclusive with the participation of patients from different Southeast Asian populations, in order to build a clear picture of the efficacy and safety of NHTs in Southeast Asian patients [49].

There are, however, several challenges in executing RCTs in Southeast Asia. There is a relatively lower incidence of PCa reported in Southeast Asian countries compared to other higher-income Asian and Western countries, leading to a smaller study subject population [1]. Furthermore, the Southeast Asian region lacks a comprehensive regional PCa registry data, which would help in planning any regional clinical trials. Other limitations include the legal framework of each country and availability of Clinical Research Organisation networks in supporting the clinical trials, as well as affordability of the new drugs in the Southeast Asian market.

The limitations in executing RCTs contribute to significant unmet needs in the management of PCa in Southeast Asia. These unmet needs include limited resources and access to NHTs for PCa patients, attributing to the limited financial capacity in both patients as well as national healthcare systems [1]. Clinical studies conducted by the pharmaceutical industry often focus on larger markets in Asia and Western countries, limiting accessibility of the new drugs in smaller markets, which in turn limits the representation of Southeast Asian data in key pivotal trials.

Furthermore, there is also a lack of infrastructure to effectively treat PCa in certain countries. This is demonstrated by the significantly lower PCa mortality-to-incidence ratio in countries with high gross domestic product and healthcare expenditure, compared to low- to middle-income countries (LMIC) [50]. The A-CaP study has revealed that a large proportion of new PCa patients in middle-income Asian countries had metastatic disease at presentation (46.6% and 53.9% of all new cases in Indonesia and Malaysia, respectively). This is in stark contrast to high-income Asian countries such as Japan, South Korea and Singapore (10.2%, 4.5%, and 15.5% of all new cases, respectively). Efforts to improve early detection of PCa could also decrease the high mortality-to-incidence ratio observed in LMIC [1].

In addition, cancer care is expensive in the Southeast Asian region. The ACTION (ASEAN Costs in Oncology) study revealed that the one-year risks for financial catastrophe (out-of-pocket health costing ≥ 30% of annual household income) and economic hardship (inability to make necessary household payments) following a cancer diagnosis were high among Southeast Asian countries, at 50% and 35% respectively. As a result, 28% took out personal loans, and 20% turned to selling assets to cover treatment costs [50].

In the M-CaP study, only 22.5% of Malaysian metastatic PCa patients received life-prolonging treatments such as chemotherapy (30.3%) or ARATs such as AA (64.3%) or enzalutamide (5.4%) after developing biochemical progression or CRPC [50]. Most patients could not afford abiraterone (~ US$ 2800/month) or enzalutamide (~ US$ 3400/month), which is > 2 × higher than the median monthly household income of ~ US$ 1410 [50]. Only 18% of Malaysian patients could afford personal insurance coverage, particularly those in the high-income groups [50].

In the near future, we hope that more resources will be channeled to the establishment of PCa registry in Southeast Asian countries to support PCa epidemiology, prevention and treatment. This evidence-based approach will aid healthcare professionals and policy makers in improving cancer services, cancer awareness programs and PCa care guidelines. In addition, cost efficacy studies can also be carried out to analyse different interventions in terms of patient health outcomes and the economic consequences, in order to understand the value of a particular therapeutic agent in this region.

Furthermore, the outcomes of the current trials of new upcoming drugs are also highly anticipated. There are currently over 100 Phase III PCa trials in progress in the United States alone [51]. Studies of interest involve various anti-cancer agents, including agents such as poly (ADP-ribose) polymerase inhibitors (PARPi), radioligand therapy with ^177^Lu-PSMA-617, immunotherapy, oral ADT, as well as a combination of these treatments, in addition to using effective drugs in earlier disease phases such as mCNPC and nmCNPC. Prevention/early diagnosis studies and novel imaging studies for more accurate disease staging are also highly anticipated.

It is important to note that although the incidence of PCa reported in the Asian population is lower compared to Western populations, the large size of the Asian population translates to a high number of PCa patients in Asia. However, key clinical trials have typically skewed towards Western patient populations, with only a small percentage of patients of Asian heritage. In fact, the low incidence of PCa, particularly in Southeast Asian countries, may actually be due to low awareness and limited PCa screening efforts in these countries. This is evident when comparing between Singaporean and Malaysian PCa registries; Malaysians of Chinese descent have a significantly lower incidence of PCa compared to Singaporeans of Chinese descent (age-standardised incidence ratios of 7.7 [2012–2016] vs. 33.4 [2014–2018] per 100,000 population), despite the two populations sharing a common ethnic origin [52, 53]. Given the above, it is essential for clinical trials to include more patients from the Southeast Asian region, which make up a large proportion of patients with unmet needs in terms of cancer treatment and knowledge on differences in disease biology.

## Conclusions

The lack of PCa clinical trials in the Southeast Asian region hampers the ability to make a robust conclusion on any specific efficacy or safety differences that may be present in this patient population. However, clinical trial sub-analyses and RWD involving patients from the broader Asian region showed a similar trend in the efficacy and safety of NHTs as the respective landmark studies. Currently, clinicians in Southeast Asia must assume that the Asian sub-analyses mirror the outcomes in this region. Expansion of future clinical trials to Southeast Asia would increase understanding on how hormonal therapies impact the diverse population of PCa patients in Southeast Asia.

## Data Availability

Data sharing is not applicable to this article as no datasets were generated or analysed in this review article.

## Abbreviations

AA: Abiraterone acetate
AAP: Abiraterone acetate plus prednisolone
ADT: Androgen deprivation therapy
AE: Adverse event
AR: Androgen receptor
ARAT: AR-axis targeted therapy
ARE: Androgen-response element
BBB: Blood–brain barrier
bPFS: Biochemical progression-free survival
CNS: Central nervous system
CRPC: Castration-resistant PCa
C_SS, ENZ_: Steady-state trough plasma concentrations of enzalutamide
C_SS, NDE_: Steady-state trough plasma concentrations of N-desmethyl enzalutamide
ECOG PS: European Cooperative Oncology Group Performance Status
HR: Hazard ratio
IQR: Interquartile range
LMIC: Low- to middle-income countries
mCNPC: Metastatic castration-naive PCa
mCRPC: Metastatic castration-resistant PCa
MFS: Metastasis-free survival
NDE: N-desmethyl enzalutamide
NHT: Novel hormonal therapy
nmCRPC: Nonmetastatic castration-resistant PCa
OS: Overall survival
PCa: Prostate cancer
PSA: Prostate-specific antigen
PFS: Progression-free survival
RCT: Randomised controlled trial
rPFS: Radiographic progression-free survival
RWD: Real-world data

## References

[1] Lim J, Onozawa M, Saad M, Ong TA, A‐CaP (Asian Prostate Cancer) Study, J‐CaP (Japan Prostate Cancer Study Group), et al. Recent trend of androgen deprivation therapy in newly diagnosed prostate cancer patients: comparing between high‐and middle‐income Asian countries. Cancer Sci 2021;112(6):2071–80. 10.1111/cas.14889 PMC8177804 33738901

[2] Health Technology Assessment Report, Ministry of Health Malaysia. Prostate cancer screening. https://www.moh.gov.my/moh/resources/auto%20download%20images/587f12cc74327.pdf. Accessed 21 Sep 2021.

[3] Sternberg CN (2012). Novel hormonal therapy for castration-resistant prostate cancer. Ann Oncol.

[4] Kim TJ, Lee YH, Koo KC (2021). Current status and future perspectives of androgen receptor inhibition therapy for prostate cancer: a comprehensive review. Biomolecules.

[5] Fizazi K, Tran N, Fein L, Matsubara N, Rodriguez-Antolin A, Alekseev BY (2019). Abiraterone acetate plus prednisone in patients with newly diagnosed high-risk metastatic castration-sensitive prostate cancer (LATITUDE): final overall survival analysis of a randomised, double-blind, phase 3 trial. Lancet Oncol.

[6] Fizazi K, Shore N, Tammela TL, Ulys A, Vjaters E, Polyakov S (2020). Nonmetastatic, castration-resistant prostate cancer and survival with darolutamide. New Engl J Med.

[7] Ryan CJ, Smith MR, Fizazi K, Miller K, Mulders P, Sternberg CN (2014). Final overall survival (OS) analysis of COU-AA-302, a randomized phase 3 study of abiraterone acetate (AA) in metastatic castration-resistant prostate cancer (mCRPC) patients (pts) without prior chemotherapy. Ann Oncol.

[8] Armstrong AJ, Iguchi T, Azad AA, Szmulewitz RZ, Holzbeierlein J, Villers A (2021). LBA25 Final overall survival (OS) analysis from ARCHES: A phase III, randomized, double-blind, placebo (PBO)-controlled study of enzalutamide (ENZA)+ androgen deprivation therapy (ADT) in men with metastatic hormone-sensitive prostate cancer (mHSPC). Ann Oncol.

[9] ESMO 2021. Final overall survival analysis from ARCHES: a phase 3, randomized, double-blind, placebo-controlled study of enzalutamide + ADT in men with mHSPC. https://www.urotoday.com/conference-highlights/esmo-2021/esmo-2021-prostate-cancer/132209-esmo-2021-lba25-final-overall-survival-os-analysis-from-arches-a-phase-3-randomized-double-blind-placebo-pbo-controlled-study-of-enzalutamide-enza-androgen-deprivation-therapy-adt-in-men-with-metastatic-hormone-sensitive-prostate-cancer-mhspc.html. Accessed 10 Nov 2021.

[10] Sternberg CN, Fizazi K, Saad F, Shore ND, De Giorgi U, Penson DF (2020). Final overall survival (OS) from PROSPER: a phase III, randomized, double-blind, placebo (PBO)-controlled study of enzalutamide (ENZA) in men with nonmetastatic castration-resistant prostate cancer (nmCRPC). J Clin Oncol.

[11] Armstrong AJ, Lin P, Tombal B, Saad F, Higano CS, Joshua AM (2020). Five-year survival prediction and safety outcomes with enzalutamide in men with chemotherapy-naive metastatic castration-resistant prostate cancer from the PREVAIL trial. Eur Urol.

[12] Chi KN, Chowdhury S, Bjartell A, Chung BH, Pereira de Santana Gomes AJ, Given R (2021). Apalutamide in patients with metastatic castration-sensitive prostate cancer: final survival analysis of the randomized, double-blind, phase III TITAN study. J Clin Oncol.

[13] Smith MR, Saad F, Chowdhury S, Oudard S, Hadaschik BA, Graff JN (2021). Apalutamide and overall survival in prostate cancer. Eur Urol.

[14] Isaacsson Velho P, Tofani Sant’Anna P, Pereira da Silva RF, Dal Ponte Ferreira R, Venero FC (2021). The development of apalutamide for the treatment of prostate cancer. Expert Opin Drug Discov.

[15] Mohler ML, Sikdar A, Ponnusamy S, Hwang DJ, He Y, Miller DD (2021). An overview of next-generation androgen receptor-targeted therapeutics in development for the treatment of prostate cancer. Int J Mol Sci.

[16] Furr BJ, Tucker H (1996). The preclinical development of bicalutamide: pharmacodynamics and mechanism of action. Urology.

[17] Rice MA, Malhotra SV, Stoyanova T (2019). Second-generation antiandrogens: from discovery to standard of care in castration resistant prostate cancer. Front Oncol.

[18] Stein MN, Goodin S, DiPaola RS (2012). Abiraterone in prostate cancer: a new angle to an old problem. Clin Cancer Res.

[19] European Medicines Agency. Assessment report—abiraterone acetate. https://www.ema.europa.eu/en/documents/assessment-report/abiraterone-accord-epar-public-assessment-report_en.pdf. Accessed 9 Nov 2021.

[20] Lim J, Amantakul A, Shariff N, Lojanapiwat B, Alip A, Ong TA (2020). Clinical outcomes of abiraterone acetate and predictors of its treatment duration in metastatic castration-resistant prostate cancer: real-world experience in the Southeast Asian cohort. Cancer Med.

[21] Fan L, Dong B, Chi C, Wang Y, Gong Y, Sha J (2018). Abiraterone acetate for chemotherapy-naive metastatic castration-resistant prostate cancer: a single-centre prospective study of efficacy, safety, and prognostic factors. BMC Urol.

[22] Chan J, Yap SY, Fong YC, Lim HC, Toh CK, Ng QS (2020). Real-world outcome with abiraterone acetate plus prednisone in Asian men with metastatic castrate-resistant prostate cancer: the Singapore experience. Asia Pac J Clin Oncol.

[23] Poon DM, Chan K, Lee SH, Chan TW, Sze H, Lee EK (2016). Abiraterone acetate in metastatic castration-resistant prostate cancer–the unanticipated real-world clinical experience. BMC Urol.

[24] Oyman A, Başak M, Özçelik M, Özyükseler DT, Işık S, Yıldırım ME (2021). Efficacy of abiraterone acetate in castration-resistant metastatic prostate cancer: a real-world data analysis. Asia Pac J Clin Oncol.

[25] Szmulewitz RZ, Peer CJ, Ibraheem A, Martinez E, Kozloff MF, Carthon B (2018). Prospective international randomized phase II study of low-dose abiraterone with food versus standard dose abiraterone in castration-resistant prostate cancer. J Clin Oncol.

[26] Saad F, Hamilou Z, Lattouf JB (2021). A drug safety evaluation of enzalutamide to treat advanced prostate cancer. Expert Opin Drug Saf.

[27] Laccetti AL, Morris MJ, Kantoff PW (2020). A clinical evaluation of enzalutamide in metastatic castration-sensitive prostate cancer: guiding principles for treatment selection and perspectives on research. Onco Targets Ther.

[28] Jung SI, Kim MS, Jeong CW, Kwak C, Hong SK, Kang SH (2020). Enzalutamide in chemotherapy-naive patients with metastatic castration-resistant prostate cancer: a retrospective Korean multicenter study in a real-world setting. Investig Clin Urol.

[29] Hori A, Sahashi H, Sano S, Matsumiya E, Ariga M, Asano A (2020). Efficacy and safety of enzalutamide in a real-world cohort of Japanese patients with castration-resistant prostate cancer. Anticancer Res.

[30] Poon DM, Wong KC, Chan TW, Law K, Chan K, Lee EK (2018). Survival outcomes, prostate-specific antigen response, and tolerance in first and later lines of enzalutamide treatment for metastatic castration-resistant prostate cancer: a real-world experience in Hong Kong. Clin Genitourin Cancer.

[31] Ji C, Guha M, Zhu X, Whritenour J, Hemkens M, Tse S (2019). Enzalutamide and apalutamide: in vitro chemical reactivity studies and activity in a mouse drug allergy model. Chem Res Toxicol.

[32] Bergström CA, Larsson P (2018). Computational prediction of drug solubility in water-based systems: qualitative and quantitative approaches used in the current drug discovery and development setting. Int J Pharm.

[33] Clegg NJ, Wongvipat J, Joseph JD, Tran C, Ouk S, Dilhas A (2012). ARN-509: a novel antiandrogen for prostate cancer treatment. Cancer Res.

[34] Bastos DA, Antonarakis ES (2019). Darolutamide for castration-resistant prostate cancer. Onco Targets Ther.

[35] Zurth C, Sandmann S, Trummel D, Seidel D, Gieschen H (2018). Blood-brain barrier penetration of [14C] darolutamide compared with [14C] enzalutamide in rats using whole body autoradiography. J Clin Oncol.

[36] Suzuki H, Shin T, Fukasawa S, Hashine K, Kitani S, Ohtake N (2020). Efficacy and safety of abiraterone acetate plus prednisone in Japanese patients with newly diagnosed, metastatic hormone-naive prostate cancer: final subgroup analysis of LATITUDE, a randomized, double-blind, placebo-controlled, phase 3 study. Jpn J Clin Oncol.

[37] Fukasawa S, Suzuki H, Kawaguchi K, Noguchi H, Enjo K, Tran N (2018). Efficacy and safety of abiraterone acetate plus prednisone in Japanese patients with newly diagnosed, metastatic hormone-naive prostate cancer: a subgroup analysis of LATITUDE, a randomized, double-blind, placebo-controlled, phase 3 study. Jpn J Clin Oncol.

[38] Iguchi T, Kimura G, Fukasawa S, Suzuki H, Uemura H, Nishimura K (2021). Enzalutamide with androgen deprivation therapy in Japanese men with metastatic hormone-sensitive prostate cancer: a subgroup analysis of the phase III ARCHES study. Int J Urol.

[39] De Giorgi U, Hussain MH, Shore ND, Fizazi K, Tombal B, Penson DF (2021). PROSPER subgroup analysis by age and region: overall survival and safety in men with nonmetastatic castration-resistant prostate cancer receiving androgen deprivation therapy plus enzalutamide. J Clin Oncol.

[40] Kim CS, Choi YD, Lee SE, Lee HM, Ueda T, Yonese J (2017). Post hoc analyses of East Asian patients from the randomized placebo-controlled PREVAIL trial of enzalutamide in patients with chemotherapy-naive, metastatic castration-resistant prostate cancer. Medicine.

[41] Chung BH, Huang J, Ye ZQ, He DL, Uemura H, Arai G (2021). Apalutamide for patients with metastatic castration-sensitive prostate cancer in East Asia: a subgroup analysis of the TITAN trial. Asian J Androl.

[42] Uemura H, Arai G, Uemura H, Suzuki H, Iijima K, Nishimura K (2021). Apalutamide for metastatic, castration-sensitive prostate cancer in the Japanese population: a subgroup analysis of the randomized, double-blind, placebo-controlled phase 3 TITAN study. Int J Urol.

[43] Graff JN, Saad F, Hadaschik BA, Mainwaring PN, Uemura H, Chowdhury S, et al. Metastasis-free survival (MFS) in nonmetastatic castration-resistant prostate cancer (nmCRPC) patients (pts) with prostate-specific antigen (PSA) decline to < 0.2 ng/ml following apalutamide (APA) treatment: post hoc results from the phase 3 SPARTAN study. Abstract MP34–20. J Urol. 2019;201:e503.

[44] Mainwaring P, Small E, Uemura H, Lee JY, Pang ST, Marx G (2018). Efficacy and safety of apalutamide (APA) in patients (pts) with nonmetastatic castration-resistant prostate cancer (nmCRPC) from SPARTAN: Asian subpopulation. Ann Oncol.

[45] Uemura H, Satoh T, Tsumura H, Arai G, Imanaka K, Shibayama K (2020). Efficacy and safety of apalutamide in Japanese patients with nonmetastatic castration-resistant prostate cancer: a subgroup analysis of a randomized, double-blind, placebo-controlled, phase-3 study. Prostate Int.

[46] Fizazi K, Shore ND, Tammela T, Ulys A, Vjaters E, Polyakov S (2019). ARAMIS: efficacy and safety of darolutamide in nonmetastatic castration-resistant prostate cancer (nmCRPC). J Clin Oncol.

[47] Chiong E, Murphy DG, Akaza H, Buchan NC, Chung BH, Kanesvaran R (2019). Management of patients with advanced prostate cancer in the Asia Pacific region:‘real-world’ consideration of results from the Advanced Prostate Cancer Consensus Conference (APCCC) 2017. BJU Int.

[48] Petracci F, Ghai C, Pangilinan A, Suarez LA, Uehara R, Ghosn M (2021). Use of real-world evidence for oncology clinical decision making in emerging economies. Future Oncol.

[49] Lim J, Malek R, Toh CC, Sundram M, Woo SY, Yusoff NA (2021). Prostate cancer in multi-ethnic Asian men: real-world experience in the Malaysia Prostate Cancer (M-CaP) study. Cancer Med.

[50] The ACTION Study Group, Bhoo-Pathy N, Yip CH, Peters SA, Kimman M, Sullivan R, et al. Policy and priorities for national cancer control planning in low- and middle-income countries: lessons from the Association of Southeast Asian Nations (ASEAN) Costs in Oncology prospective cohort study. Eur J Cancer. 2017:74:26–37. 10.1016/j.ejca.2016.12.014 28335885

[51] Prostate Cancer Foundation. Prostate cancer clinical trials. https://www.pcf.org/patient-resources/patient-navigation/prostate-cancer-clinical-trials/. Accessed 6 Jan 2022.

[52] Ministry of Health Malaysia. Malaysia National Cancer Registry Report 2012–2016. https://www.moh.gov.my/moh/resources/Penerbitan/Laporan/Umum/2012-2016%20(MNCRR)/MNCR_2012-2016_FINAL_(PUBLISHED_2019).pdf. Accessed 9 Feb 2022.

[53] Singapore Health Promotion Board. Singapore Cancer Registry Annual Report 2018. https://www.nrdo.gov.sg/docs/librariesprovider3/default-document-library/scr-annual-report-2018.pdf?sfvrsn=bcf56c25_0. Accessed 9 Feb 2022.

[54] PubChem. Abiraterone acetate. https://pubchem.ncbi.nlm.nih.gov/compound/Abiraterone-acetate#section=Structures. Accessed 9 Nov 2021.

[55] PubChem. Enzalutamide. https://pubchem.ncbi.nlm.nih.gov/compound/Enzalutamide. Accessed 20 Oct 2021.

[56] PubChem. Apalutamide. https://pubchem.ncbi.nlm.nih.gov/compound/Apalutamide. Accessed 20 Oct 2021.

[57] PubChem. Darolutamide. https://pubchem.ncbi.nlm.nih.gov/compound/Darolutamide. Accessed 13 Oct 2021.

[58] European Medicines Agency. Assessment report For Zytiga (abiraterone). https://www.ema.europa.eu/en/documents/assessment-report/zytiga-epar-public-assessment-report_en.pdf. Accessed 9 Nov 2021.

[59] Center for Drug Evaluation and Research. Application number- 203415Orig1s000: clinical pharmacology and biopharmaceutics review. https://www.accessdata.fda.gov/drugsatfda_docs/nda/2012/203415Orig1s000ClinPharmR.pdf. Accessed 8 Feb 2022.

[60] Product monograph. ERLEADA™. https://pdf.hres.ca/dpd_pm/00046215.PDF. Accessed 5 Jan 2022.

[61] Product monograph. ZYTIGA™. https://pdf.hres.ca/dpd_pm/00044223.PDF. Accessed 5 Jan 2022.

[62] Product monograph. XTANDI™. https://www.astellas.com/ca/system/files/pdf/Xtandi_PM_EN.pdf. Accessed 5 Jan 2022.

[63] Product monograph. NUBEQA™. https://www.bayer.com/sites/default/files/2020-11/nubeqa-pm-en.pdf. Accessed 5 Jan 2022.

